# Application of a double reverse traction repositor in the retrograde intramedullary nailing of distal femur fractures

**DOI:** 10.1186/s13018-021-02324-6

**Published:** 2021-03-03

**Authors:** Xiaodong Lian, Kuo Zhao, Wei Chen, Junzhe Zhang, Junyong Li, Hongyu Meng, Zhiyong Hou, Yingze Zhang

**Affiliations:** 1 Department of Orthopaedic Surgery, Third Hospital of Hebei Medical University, No. 139 Ziqiang Road, Shijiazhuang, 050051 Hebei PR China; 2 Key Laboratory of Biomechanics of Hebei Province, Shijiazhuang, Hebei 050051 PR China; 3 Orthopaedic Research Institution of Hebei Province, Shijiazhuang, Hebei 050051 PR China; 4 NHC Key Laboratory of Intelligent Orthopaedic Equipment, The Third Hospital of Hebei Medical University, Shijiazhuang, China; 5 Chinese Academy of Engineering, Beijing, 10088 P.R. China

**Keywords:** Distal femur fractures, Retrograde intramedullary nailing, Traction technique, Double reverse traction repositor

## Abstract

**Objective:**

The purpose of this prospective study was to introduce the application of a double reverse traction repositor (DRTR) in the retrograde intramedullary nailing (RE-IMN) of AO/OTA 33A distal femur fractures.

**Patients and methods:**

A total of 27 patients with AO/OTA type 33A distal femur fractures who were admitted from January 2015 to May 2017 to a level I trauma center of a tertiary university hospital were enrolled in this prospective study. A DRTR was used to facilitate RE-IMN for the reduction of distal femur fractures in all patients. The demographic and fracture characteristics, surgical data, postoperative complications, and prognostic indicators of 24 patients were recorded.

**Results:**

The DRTR helped achieve and maintain the reduction of all distal femur fractures in the present study. All surgeries were conducted by closed reduction, and excellent alignment was observed in the postoperative X-ray images. In the present study, 18 males and 6 females were included, and the average age of all patients was 51.3 years (range, 24–68 years). The mean operation time, intraoperative blood loss, intraoperative fluoroscopy time, and length of postoperative hospital stay were 137 min (range from 80 to 210 min), 320 ml (range from 200 to 600 ml), 28 (from 24 to 33), and 9 days (from 5 to 14 days), respectively. Eleven patients were found to have postoperative deep venous thrombosis before discharge. No cases of wound infection were observed. No cases of nonunion or malunion were observed. The average follow-up duration was 21 months (18–30 months). The average HHS, LKFS, and VAS scores at the 1-year follow-up were 89.9 (86–97), 79.1 (75–87), and 2.1 (from 0 to 5). No complications associated with DRTR were found.

**Conclusions:**

A DRTR can be successfully applied in the treatment of distal femur fractures with RE-IMN, and it can not only help achieve or maintain the reduction of distal femur fractures with closed methods but also promote fixation with RE-IMN.

## Introduction

Distal femur fractures are uncommon, and the associated morbidity and mortality rates are underestimated [1]. Distal femur fractures comprise 4–8% of all femur fractures, and incidence of these fractures is approximately 18/100,000 person-years [2, 3]. A bimodal age distribution has been found in the occurrence of distal femur fractures, with one peak in young male patients with high-energy injuries and the other in geriatric female patients with low-energy injuries [1]. Due to population aging worldwide, the incidence of distal femur fractures is expected to increase further. Distal femur fractures are still challenging for orthopaedic trauma surgeons to treat. Intramedullary nailing (IMN) and locking plate systems have gradually become widely used for the surgical treatment of distal femur fractures, with advancements in minimally invasive strategies [4]. However, no consensus has been reached among surgeons on the best way to treat distal femur fractures, especially extra-articular distal femoral fractures [4–6]. Retrograde intramedullary nailing (RE-IMN) has been proven to be an efficient technique for the management of distal femur fractures [7]. The indications are extra-articular fractures and simple intra-articular fractures, such as AO/OTA 33A fractures [8, 9].

RE-IMN is superior to locking plate systems for treating distal femur fractures with specific patterns. It allows for less invasive insertion, minor soft tissue stripping, less intraoperative blood loss, a shorter duration of surgery, earlier mobilization, and better axial alignment consistent with the axial alignment of the femur [4, 10]. Previous studies have demonstrated that RE-IMN is comparable to locking plates in terms of the biomechanical properties in distal femur fractures with specific patterns. Du et al. compared locking plates and RE-IMN in the fixation of distal femur fractures and found that both implants can provide sufficient biomechanical stability and that RE-IMN is better than locking plates regarding the deformation of the fracture site [4]. In a biomechanical study comparing nailing and plating for distal femur fractures, Mehling et al. found comparative levels of biomechanical stability under physiological torsional and axial loading between RE-IMN and a less invasive stabilization system plate [11]. Wähnert et al. conducted a study to investigate the biomechanical stability of four different fixation devices for the treatment of comminuted distal femoral fractures in osteoporotic bone [12]. They demonstrated that the nails had a higher combined (torsional and axial) biomechanical stability than did the angular stable plates. In addition, it has been reported that better functional outcomes are observed in patients who undergo RE-IMN than in those who undergo treatment with locking plates [10, 13]. In a retrospective study comparing the long-term functional outcomes after distal femur fracture surgery fixed with locking plates or RE-IMN, Hoskins et al. discovered that RE-IMN was superior to locking plates in the functional outcomes within 1 year postoperatively [7]. They also observed a significant reduction in angular deformities by using RE-IMN. Gao et al. evaluated locking plates and RE-IMN for the treatment of extra-articular distal femoral fractures and discovered that the union rate with RE-IMN was higher than that with locking plates [14]. In a study of locking plates versus RE-IMN in the treatment of supracondylar femur fractures, Kyriakidis et al. demonstrated that RE-IMN was superior to locking plates in fracture healing [15]. More symmetric and improved callus formation was also observed in patients who underwent RE-IMN in previous studies [1, 7, 16].

One limitation of RE-IMN is that the insertion of nails cannot help reduce fractures [1]. The knee should remain in 30° flexion during the operation to facilitate RE-IMN, and thus, the traction table cannot be used in the reduction of the fractures. The traction table is a crucial tool for the reduction of lower limb fractures, as the table can provide continuous and stable traction forces to reduce fractures [17]. To date, no devices have been observed to facilitate the reduction of distal femur fractures with RE-IMN. In our previous studies, we designed a rapid redactor, a double reverse traction repositor, to help achieve and maintain the reduction of long bone fractures of limbs, including hip fractures, femur shaft fractures, and tibial plateau fractures [18–22]. Chen et al. introduced the application of DRTR in the surgical treatment of femur shaft fractures [21]. Zhang et al. conducted a study to compare the DRTR and traction table in the treatment of femur shaft fractures with anterograde IMN [20]. The authors demonstrated that the DRTR can lead to comparable or even better outcomes in the treatment of femur shaft fractures with anterograde IMN.

On the basis of the results of our previous studies, we hypothesized that the DRTR can successfully be used in the surgical treatment of distal femur fractures with RE-IMN. The purpose of this study was to (i) introduce the use of the DRTR in the surgical treatment of distal femur fractures with RE-IMN, (ii) evaluate the postoperative functional and radiological outcomes in patients treated with the DRTR, and (iii) observe whether complications associated with the application of DRTR occur.

## Patients and methods

Patients with distal femur fractures who underwent surgical treatment in our department (level I trauma center of a tertiary university hospital) were recruited for our study. The inclusion criteria of this study were patients with distal femoral fractures (AO/OTA type 33A) treated by RE-IMN between January 2015 and May 2017. The exclusion criteria were as follows: (a) patients aged < 18 years; (b) patients with multiple fractures or open fractures; (c) patients with pathological fractures or old fractures (time from injury to surgery > 21 days); (d) patients who rejected the use of the DRTR or were unable to apply the device due to poor local skin conditions; (e) non-ambulatory patients or patients who could not tolerate a surgical procedure due to frailty; and (f) patients with incomplete data. The Institutional Review Board of the Third Hospital of Hebei Medical University approved this study. Informed consent was obtained from all patients before surgery. This study was conducted following the ethical standards in the Declaration of Helsinki.

A total of 24 patients were enrolled in our study according to the inclusion and exclusion criteria. In the present study, 18 males and 6 females were included, and the average age of all patients was 51.3 years (range, 24–68 years). Essential preoperative examinations and preparation processes were conducted upon admission. The average time from admission to surgery among all patients was 3 days (range, 2–7 days) (Table 1).

**Table 1:** Demography and fracture-related characteristics

Patients	Gender	Age (years)	BMI	ASA (1=I–II, 2=III–IV)	Damage mechanism	Side
1	Male	64	25.4	2	High energy	Right
2	Female	60	28.1	2	High energy	Left
3	Male	45	25.6	1	High energy	Right
4	Male	66	24.8	2	High energy	Left
5	Female	58	25.4	2	Low energy	Right
6	Female	63	22.6	1	Low energy	Right
7	Male	26	18.3	1	High energy	Left
8	Male	85	21.8	2	High energy	Right
9	Female	32	24.3	1	High energy	Left
10	Male	33	29.7	2	High energy	Left
11	Male	51	30.2	2	Low energy	Right
12	Male	43	27.3	2	High energy	Right
13	Male	50	28.7	2	High energy	Left
14	Female	71	26.6	2	Low energy	Right
15	Male	61	29.4	2	High energy	Left
16	Female	82	29.3	2	High energy	Left
17	Male	45	24.8	1	High energy	Right
18	Male	45	25.9	1	Low energy	Left
19	Male	32	30.0	1	High energy	Left
20	Male	30	28.7	1	High energy	Left
21	Male	24	27.6	1	High energy	Left
22	Male	45	26.7	1	High energy	Right
23	Male	68	28.3	2	Low energy	Right
24	Male	53	25.6	1	Low energy	Right

### Surgical technique

**Fig. 1:**
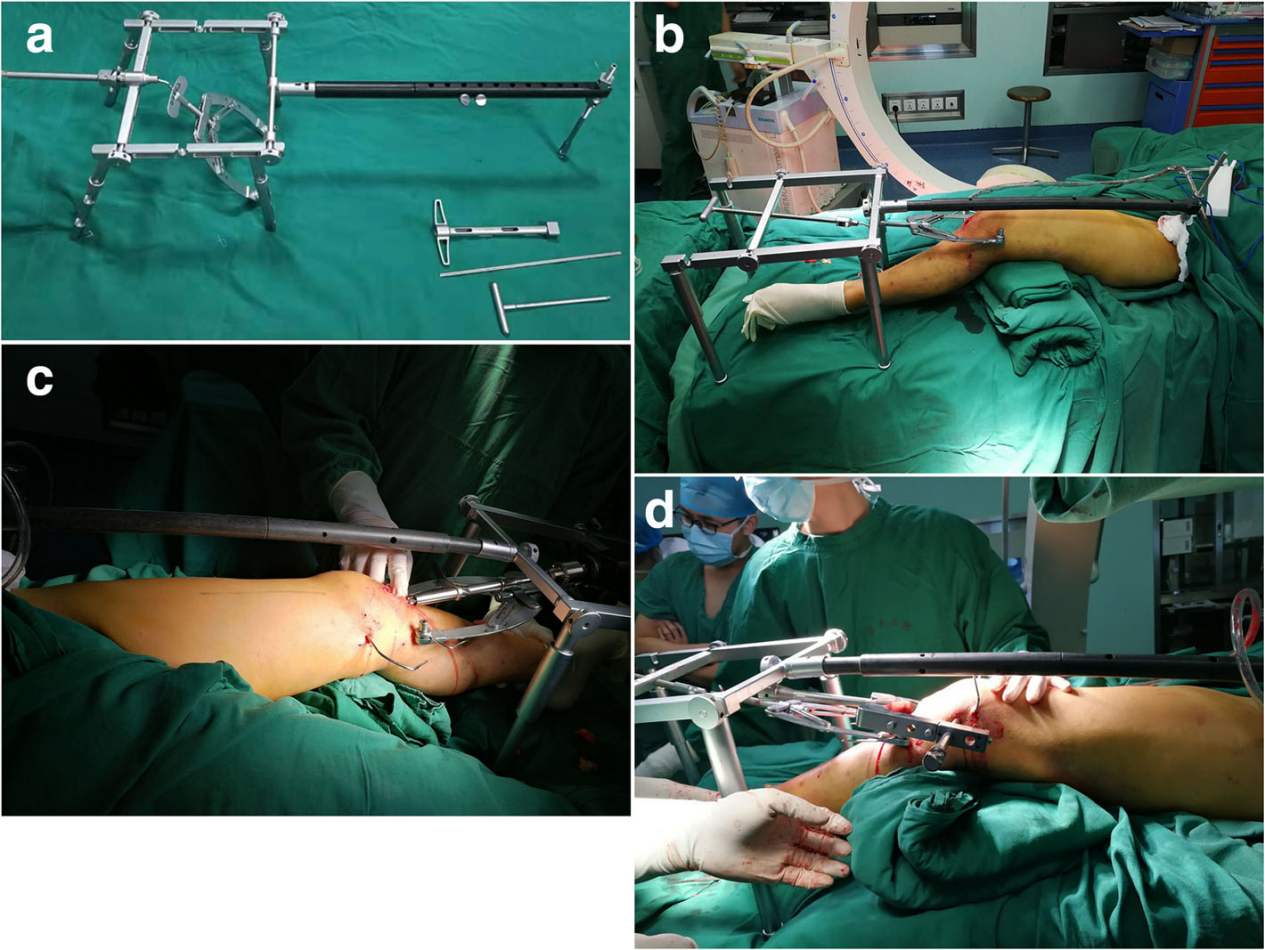
The application of DRTR was shown in **a**–**d**. **a** The lateral view of DRTR. **b** Intraoperative gross image. **c** Intraoperative reaming image. **d** Image of locking distal screw

The DRTR consists of a reduction scaffold, traction bow, connecting rod, and proximal connecting device (Fig. 1a). The distal bar of the reduction scaffold could be moved from position A to B according to the length of the patient’s thigh (Fig. 1a). In this study, all surgeries were conducted by the same team, which consisted of two orthopedic trauma surgeons with more than 8 years of experience who had been treating more than 70 distal femur fractures annually.

**Fig. 2:**
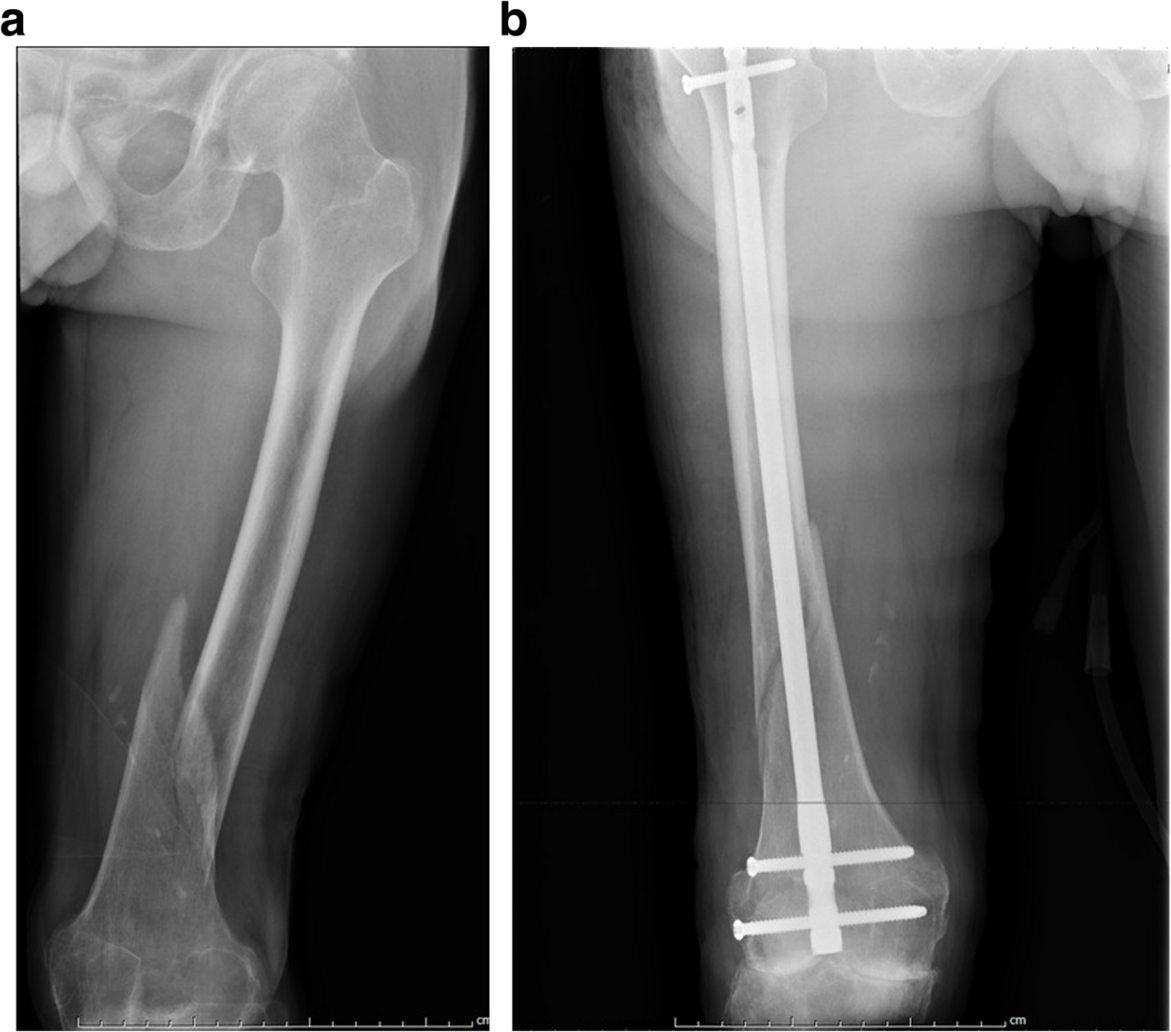
A 45-year-old patient sustained a right distal femur fracture. **a** Preoperative X-ray. **b** Postoperative X-ray

Anesthesia and the patient position were established (Step 1). All the patients were under spinal or general anesthesia before surgery. After anesthesia, the patients were placed in a supine position, and a radiolucent table was used to assist fluoroscopy during the operation. The approach, exposure, and reduction process were performed (Step 2). First, the proximal connecting device was fixed. A 2-cm incision was made around the ipsilateral anterior superior iliac spine to reveal the iliac spine. The proximal connecting device around the anterior superior iliac spine was fixed with a 5-mm screw. The screws were passed through both cortices. The upper screw of the proximal connecting device was tightened so that the proximal device made full contact with both sides of the iliac bone, which increases the area of stress between the proximal connecting device and iliac bone to prevent avulsion fractures at the fixation site. Second, the insertion site of the intramedullary nail was identified. The distal femur was supported with drapes so that the knee remained in 30° of flexion. A 4-cm longitudinal incision was made around the medial part of the patellar tendon. Excision of the infrapatellar fat pad was required to reveal the intercondylar fossa, which was the insertion site of the intramedullary nail. Third, the DRTR was installed (Fig. 1b). The proximal traction site of the DRTR was the iliac bone, and the distal traction site was at the supracondylar femur or the tubercle of the tibia. The diameters of the Kirschner wire were 3.0 mm and 2.5 mm for the supracondylar traction of the femur and traction of the tibial tubercle, respectively. Supracondylar traction of the femur was performed in most cases to produce a higher traction force. When implanting the Kirschner wire, the supracondylar articular surface of the femur was chosen as a reference and implanted as close as possible to the front of the femur so that it would not affect the insertion of the intramedullary nail. It was important to ensure that the Kirschner wire passed through two layers of cortical bone. The DRTR was installed after the preparations. Fourth, the fractures were reduced. The handle of the DRTR was rotated until the quadriceps were fully strained to produce a sufficient traction force. The reduction of the fracture was observed under fluoroscopy. If there was residual lateral displacement or anteroposterior angular displacement, the distal limb was moved to complete the reduction process. The RE-IMN was implanted (Step 3). An opening tool was used to drill holes in the distal femur. The golden finger was inserted from the distal femur to the proximal femur. Intraoperative fluoroscopy was used to ensure that the golden finger was located in the medullary cavity of the femur. A guide wire was inserted into the femur through the golden finger. Different types of reamers were used to ream the medullary cavity of the femur in turn (Fig. 1c). Then, the main nail was implanted. After inserting the distal locking nail (Fig. 1d), the DRTR was removed. Finally, the proximal screws and the tail cap were implanted. For comminuted fractures, the femur length might be shortened without the traction of the DRTR. To prevent shortening for maintaining the normal femur length, the DRTR was removed after the insertion of the proximal locked screws. And the proximal locked screws were inserted by a free-hand technique in these patients. After the positions of all the screws were confirmed by fluoroscopy (Fig. 2), the incisions were sutured (Step 4).

### Postoperative management

Routine prophylactic antibiotics were used within 24 h after surgery. All patients were injected with subcutaneous low-molecular-weight heparin sodium the first day after surgery. Rehabilitation exercises were required after surgery. Postoperative partial weight bearing was allowed 6 weeks postoperatively. Follow-ups were performed at 1, 3, 6, and 12 months postoperatively and every 6 months thereafter. The radiological and functional outcomes were evaluated at each follow-up. Full weight bearing was permitted when enough callus was observed on the X-ray image. The radiological outcomes were evaluated by X-ray, and the functional outcome measures included the Harris Hip Score (HHS) [23], Lysholm Knee Function Score (LKFS) [24], and visual analog scale (VAS) score [25].

#### Harris Hip Score

The HHS was used to evaluate postoperative hip function in the patients. The assessment tool mainly addresses 4 aspects: pain, function, the absence of deformity, and range of motion. The best possible score is 100 points. The scores were classified as poor (<70), fair (70–80), good (80–90), or excellent (90–100).

#### Visual analog scale

The VAS was used to assess the severity of pain at each follow-up and ranged from 0 (no pain) to 10 (most severe pain); the scores were considered excellent (0–2 score), good (3–5 score), fair (6–8 score), or poor (> 8 score) [25].

## Results

The DRTR helped achieve and maintain the reduction of all distal femur fractures in the present study. All surgeries were conducted by closed reduction, and excellent alignment was observed in the postoperative X-ray images.

### Surgical data

The mean operation time was 137 min (range from 80 to 210 min). The average intraoperative blood loss was 320 ml (range from 200 to 600 ml). The mean intraoperative fluoroscopy time was 28 (from 24 to 33). The mean length of postoperative hospital stay was 9 days (from 5 to 14 days) (Table 2).

**Table 2:** Surgical data

Patients	Time to surgery (days)	Anesthesia	Duration of surgery (mins)	Blood loss (ml)	Intraoperative blood transfusion (ml)	Intraoperative fluoroscopy times	Reduction methods
1	5	General	95	300	0	25	Closed
2	3	General	90	200	300	27	Closed
3	6	General	100	600	600	30	Closed
4	3	General	120	500	800	24	Closed
5	6	Spinal	135	200	300	26	Closed
6	3	General	140	300	600	28	Closed
7	3	Spinal	100	200	0	31	Closed
8	6	General	160	500	200	30	Closed
9	2	General	90	200	0	33	Closed
10	2	General	195	400	0	28	Closed
11	2	General	120	300	0	27	Closed
12	6	Spinal	180	400	0	26	Closed
13	3	General	210	500	300	29	Closed
14	3	General	80	300	300	30	Closed
15	2	General	150	300	200	31	Closed
16	1	General	80	200	0	32	Closed
17	3	Spinal	120	200	0	28	Closed
18	2	General	150	300	0	29	Closed
19	2	Spinal	170	200	200	26	Closed
20	2	General	160	200	0	22	Closed
21	3	Spinal	190	600	800	35	Closed
22	5	Spinal	180	300	800	27	Closed
23	5	Spinal	135	200	0	29	Closed
24	3	Spinal	140	300	800	28	Closed

### Prognosis data

The average follow-up was 21 months (18–30 months). The average HHS and LKFS at the 1-year follow-up were 89.9 (86–97) and 79.1 (75–87), respectively. The mean VAS was 2.1 (from 0 to 5), and the VAS results of all patents were excellent or good (Table 3).

**Table 3:** Prognostic data

Patients	Postoperative hospital stays (days)	Deep vein thrombosis	Postoperative infection	HHS	LNFS	VAS	Follow up (months)
1	12	Yes	No	92	79	2	18
2	6	Yes	No	90	81	2	24
3	10	No	No	89	82	1	18
4	10	No	No	88	84	0	18
5	8	Yes	No	91	79	2	18
6	14	No	No	93	77	3	24
7	5	No	No	92	78	4	24
8	8	No	No	90	76	4	18
9	6	Yes	No	88	77	2	24
10	13	No	No	88	78	0	18
11	11	No	No	89	79	3	24
12	5	No	No	92	80	5	18
13	13	Yes	No	88	79	3	24
14	9	Yes	No	89	78	2	18
15	10	Yes	No	90	79	0	24
16	5	No	No	88	77	2	18
17	9	Yes	No	87	81	2	24
18	9	No	No	97	78	1	18
19	12	Yes	No	88	79	3	24
20	6	Yes	No	86	76	2	18
21	12	No	No	93	80	1	18
22	13	No	No	94	75	3	30
23	6	Yes	No	88	79	0	24
24	12	No	No	88	87	2	18

*HHS* Harris Hip Score, *LNYS* Lysholm Knee Function Score, *VAS* Visual analog scale

### Postoperative complications

Eleven patients were found to have postoperative deep venous thrombosis before discharge. All of the deep venous thrombosis cases were partially recanalized after treatment with low-molecular-weight heparin. No cases of wound infection were discovered. No cases of nonunion or malunion were observed in this study. No complications associated with DRTR were found in this study.

## Discussion

In this study, the DRTR was applied to facilitate the reduction of distal femur fractures, and we found that it could help achieve and maintain reduction in the retrograde intramedullary nailing of distal femur fractures. Meanwhile, all patients obtained satisfactory results in terms of radiological outcomes or functional outcomes.

### The necessity of the application of DRTR in RER-IMN

Although the choice of implants has made progress, the ideal implants in the treatment of distal femur fractures [7] remain controversial. Modern locking plates have been proven to be an excellent option for the treatment of distal femur fractures, but they are often associated with a high nonunion rate, metalwork failure rate, and delayed union rate [16, 26]. RE-IMN is a treatment option involving minimally invasive insertion for distal femur fractures, especially AO/OTA type 33A fractures. Different from antegrade IMN, for which a traction table can be used to facilitate the reduction of fractures, no devices can be used to help achieve the reduction of distal femur fractures with RE-IMN. Therefore, open reduction is usually required for patients with irreducible distal femoral fractures that cannot be treated with closed reduction with intramedullary nails. In addition, it has been reported that the insertion of nails cannot facilitate the reduction of fractures with metaphyseal extension, and fractures must be reduced in the coronal and sagittal planes before reaming and the insertion of nails. Otherwise, malreduction may occur [27, 28].

### The advantages of the DRTR

Similar to the traction table, the DRTR can provide continuous and adequate traction forces for the reduction of fractures. Chen et al. and Zhang et al. demonstrated that the DRTR can provide enough force to achieve the reduction of femur shaft fractures. Our previous study proved that the DRTR can correct displaced intertrochanter fractures. In the present study, all the surgeries were conducted by closed reduction with the DRTR, and excellent alignment was observed in the postoperative X-ray images. Moreover, no complications associated with the application of the DRTR were found in this study. With the DRTR, the reduction time may decrease, and the duration of surgery or anesthesia may decrease as well. A shorter duration of surgery or anesthesia reduces the risk of postoperative complications [29]. Compared to manual traction, the use of the DRTR can reduce the number of assistants needed, which is important to control the medical costs and decrease the wound infection rate [30, 31]. The mean operation time was 137 min (range from 80 to 210 min) in the present study. Although the installation of the DRTR takes extra time, the time needed for reduction decreases. Therefore, the duration of surgery or anesthesia does not increase. In addition, the orientation of distal traction was vertical. Supracondylar traction of the femur was selected in most cases, especially for those in which the distal end of the fracture was displaced angularly or shortened posteriorly. Traction of the tibial tubercle is more suitable for fractures in which the distal end of the fractures is displaced angularly or shortened anteriorly. Lastly, maintaining the reduction was difficult in the insertion of proximal locked screws for the comminuted fractures by manual traction, while the DRTR could be used to maintain the normal femur length at the insertion of the proximal locked screws. Although the DRTR might be not conducive to the insertion of the proximal locked screws, a free-hand technique could be used to achieve the insertion of the proximal locked screws in this study.

The diagnosis of DVT was made by sonographers through Colour Doppler ultrasound, which was used to detect the presence of DVT in both lower limbs at hospitalization, after the operation (1–2 days after surgery), and every 3–5 days postoperatively until discharge. A total of 11 patients were found with postoperative DVT in this study, of which 8 patients were from preoperative DVT and 3 patients were postoperative new-onset DVT. The DVT included 9 calf muscular vein thrombosis, 1 posterior tibial DVT, and 1 peroneal vein DVT in this study. The inclusion of calf muscular vein thrombosis and the small sample size might be associated with the high incidence of DVT.

### Limitations

There were some limitations of the present study. First, the sample size in this study was small. Second, we did not enrol patients with distal femur fractures treated by manual traction, and a comparison between the DRTR and manual traction in the treatment of distal femur fractures could not be conducted. Therefore, large-scale and multicenter research should be conducted in the future to compare the results of the DRTR and manual traction in the treatment of distal femur fractures.

## Conclusion

The DRTR could be applied in the treatment of distal femur fractures with RE-IMN. The DRTR not only helped achieve or maintain the reduction of distal femur fractures with closed methods but also facilitated fixation with RE-IMN.

## Data Availability

The datasets used and/or analyzed during the current study are available from the corresponding author upon reasonable request.

## Abbreviations

DRTR: Double reverse traction repositor
RE-IMN: Retrograde intramedullary nailing
IMN: Intramedullary nailing
HHS: Harris Hip Score
LKFS: Lysholm knee function score
VAS: Visual analog scale
